# Development and validation of a Social Images Evaluation Questionnaire for youth in residential care

**DOI:** 10.1371/journal.pone.0179890

**Published:** 2017-06-29

**Authors:** Diniz Lopes, Maria Manuela Calheiros, Joana Nunes Patrício, Margarida Vaz Garrido

**Affiliations:** Instituto Universitário de Lisboa (ISCTE-IUL), CIS-IUL, Lisboa, Portugal; Public Library of Science, FRANCE

## Abstract

Social images are defined as prevailing shared ideas about specific groups or societies without concrete or objective evidence of their accuracy or truthfulness. These images frequently have a negative impact on individuals and groups. Although of outmost importance, the study of the social images of youth in residential care is still scarce. In this article we present two studies for the development and validation of the Social Images Evaluation Questionnaire (SIEQ). In study 1, participants were asked to freely generate words that could be associated to youth in residential care in order to obtain a list of attributes to be used in the SIEQ. In study 2, the main psychometric characteristics of the SIEQ were tested with samples of laypeople and professionals. The main results support the proposal of a new and psychometrically sound measurement–the SIEQ–to analyze the social images of youth in residential care.

## Introduction

Social images are normally defined as prevailing shared ideas regarding specific groups or societies without concrete or objective evidence of their accuracy or truthfulness [1]. They constitute shared social beliefs about the characteristics (i.e., personality traits, behaviors, values) of specific social groups and its members, independently of their veracity [1,2,3]. In the present article, we propose a measure for tapping the social images of youth in residential care.

As shared beliefs, social images can be positive or negative emerging from shared personal experiences, external influences exerted by different contexts where individuals interact (e.g., family, community, job), or social media (e.g., books, television, radio, press) [2,4]. Negative social images are potentially more stigmatizing and can affect individuals through processes of expectancy confirmation, discrimination, negative interactions, automatic activation of stereotypes, or even identity threats [5]. Indeed, social images have been associated to perceived low quality of life and well-being, stress, depression, fear, low self-esteem, unemployment, health problems, and psychological distress, among others (e.g., [5,6,7,8,9,10]). Also, perceived discrimination has been shown to impact on mental illness by increasing symptoms of depression, anxiety, psychosis or paranoia [11,12]. The impact of social images may as well vary according to personal characteristics of individuals and their coping strategies (e.g., stigma) [5]. Social images also play an important role in individual development of identity [13,14,15,16]. Therefore, stereotypical images are particularly important in adolescence, a time of self-construction and reconstruction [17] towards the consolidation of identity (e.g., [18]). Hence, internalization of negative social images during this period may have a particularly negative impact on adolescents’ process of identity construction.

Studies on social images have been focusing mainly on gender, age, and ethnic-related factors (e.g., cultural differences). However, in the last decades research in this area has been enlarging its focus [19]. Although still underdeveloped, one of the emerging research areas in this domain has been the study of social images of youth in residential care. In this study, residential care is conceptualized as an institutional care service with facilities, equipment, and technical staff required for permanent care of children and youth in order to guarantee their education, wellbeing, and development. To our knowledge, no studies have so far presented instruments that examine the social image of youth in residential care. In this article we present the development and validation of a questionnaire examining social images associated with youth in residential care–the Social Images Evaluation Questionnaire (SIEQ).

### The social images of youth in residential care

The few studies addressing the social images of youth in residential care indicate that they are perceived in a negative way, or are target of a negative social image [20,21,22]. Indeed, the results of discriminant analyses reported by Calheiros et al. [20] indicate that children and youth in residential care were mainly described with negative attributes, while residential care institutions were mainly described with positive attributes. Moreover, the results also suggested that these social images are consensually shared in society, since they did not vary according to professional contact with this population (i.e., laypeople vs. residential care professionals), nor according to gender, age or educational level of the respondents.

The social images of children and youth in residential care include different dimensions, namely behavioral (e.g., aggressive, individuals in risk, marginal, problematic, hostile, contemptuous), emotional (e.g., deprived, sad, rebel, sensitive), social (e.g., abandoned, lone, introverted), physical (e.g., dirty), cognitive, scholar or professional (e.g., insecure, failed, unqualified, with weak academic competences), and economical (e.g., poor) [20,21,22,23,24,25]. Institutionalization also appears associated with a negative social image, which in turn exerts a strong impact on well-being and identity of youth in residential care [21]. For example, a study by Simsek, Erol, Öztop, and Münir [26] showed that feelings of stigmatization among individuals in residential care units were associated with behavioral and emotional problems. Thus, the study of the social images in this context is extremely important both for advancing the literature in this area and for the potential applications that these studies are likely to have for professionals working with youth, for society in general, and more importantly for the quality of life and well-being of institutionalized youth.

However, the available studies in this domain analyze the social image of youth in residential care mainly by making use of qualitative methods such as individual interviews (e.g., [24]), focus-groups (e.g., [27,28]), life history (e.g., [23]), and questionnaires with open-ended questions (e.g., asking individuals to generate words associated to youth and to residential care) [20,21]. These methods can be highly advantageous in allowing spontaneous and unconstrained answers from the respondents, and in unveiling often-unexpected social images. However, they do not allow the quantification of the attributes that are most descriptive of different groups, creating difficulties in the systematic and comparative study of social images of youth in residential care. Other methods also utilize true-false or agree-disagree Likert-type questionnaires with items reflecting laypeople’s common social images (e.g., [29]).

Importantly, no study has to our knowledge adopted a more systematic approach for examining the social image of youth with both qualitative and quantitative approaches. This article presents a scale developed with this twofold methodological approach, that is, a qualitative approach to the social images of youth in residential care is complemented with another of quantitative nature. Following this rationale, in this article we present two studies aiming at developing a measure to evaluate the social images of youth in residential care–the SIEQ. In the first study, and following the methodology of Zafar and Ross [30] and Martins and Calheiros [31], we asked participants to freely generate words that could be associated to youth in residential care, in order to obtain a list of attributes to be used in a subsequent questionnaire. In the second study, we present the development of the SIEQ and the test of the scale’s main psychometric characteristics.

## Study 1

### Overview

The goal of this first study was to obtain a list of words related to youth in residential care to be used in the development of a questionnaire to measure the social images of youth in residential care—the SIEQ. To this end, and following the methodology of Zafar and Ross [30] and Martins and Calheiros [31], as well as on the adjective checklist methodology proposed by Katz and Braly [32], we asked participants to freely generate words that could be associated to youth in residential care and mainstream youth.

### Method

#### Participants

One hundred seventy-six participants freely agreed to answer a questionnaire regarding children and youth in residential care. Participants age varied between 18 and 77 years old (*M* = 34.60, *SD* = 14.02), 80.6% were female, 59.5% were single, 35.3% were married or cohabiting couples, 4% were divorced, and 1.2% were widowers. From these, 36% had one to three children aged between 0 to 37 years old (*M* = 16.36, *SD* = 9.54). The majority of the participants (57.1%) completed a major, 32.9% completed high school and 10% elementary school. For 43.6% of the participants the family income fell between 1000 and 2000 euros per month, while for 21.4% this income was below 1000 euros. The remaining participants revealed that their monthly family income was above 2000 euros. About one third (34.3%) of the participants revealed that they knew at least one child/youth in residential care, and 13.7% of them worked in the area of children/youth at risk or danger.

#### Instruments

Using an open-ended questionnaire, we asked participants to generate five attributes/characteristics that they would normally associate to mainstream youth (between 12 and 18 years old), and another five normally associated to youth (between 12 and 18 years old) in residential care (counterbalanced). In the beginning of the questionnaire, and before generating the attributes for youth in residential care, participants could read “Residential care constitutes one of the services aiming to protect and safeguard the fundamental rights of children and youth who, in their natural living environments, are exposed to conditions prejudicial to their development. This institutional care service involves the placement of children and youth in the care of an entity with facilities and equipment required for permanent care, and a technical team that guarantees care in accordance with their needs, in order to provide the conditions enabling their education, wellbeing, and integral development [legal definition of residential care, *Diário da República*, (Portuguese Official Gazette), Law 147/99, 1^st^ September] [33]. Please, think of youth aged between 12 and 18 years old living in residential care. How would you describe this youth? Write down five characteristics / attributes of a youngster living in such a context.”

For mainstream youth participants read “Please think of youth aged between 12 and 18 years old that lives with his/her family. How would you describe this youth? Please write down five characteristics / attributes of a youngster living in such a context”

In a second section of the questionnaire, we asked participants if they worked with youth at risk to differentiate care sector professionals from laypeople. Afterwards, participants answered a set of sociodemographic questions, namely age, gender, educational qualifications, average monthly income, and number and age of children of their own.

#### Procedure

Participants were recruited through convenience and snowball sampling, that is, according to their accessibility and proximity to the researchers. Professionals were recruited from care institutions, and protection services for children and youth. Specifically, we contacted the Directors of several care institutions and youth protection services in Portugal to present the study, and to ask their permission to collect data from the staff of their institutions. After their approval, the study was presented to the staff via email and those who agreed to participate were asked to collect a questionnaire at the administrative office of the institution. A similar procedure was followed regarding laypersons recruited from teaching and training institutions.

Data was collected via a paper and pencil questionnaire delivered in a closed envelope. At the beginning of the questionnaire, participants were told that the aim of the study was to collect their opinions about the characteristics or attributes of hypothetical youth in residential care and mainstream youth. It was highlighted that there were no right or wrong answers and that all data collected was confidential and anonymous. Participants were further assured that data would be analyzed as a whole. In the end, participants were debriefed and thanked for their collaboration, and were asked to return the questionnaire to the administrative office of their institution.

The study was conducted in accordance with the existing Ethical Guidelines at Instituto Universitário de Lisboa (ISCTE-IUL), and approved by the Board of the Ethical Commission of the same institution.

### Results

In a first stage, the overall attributes listed by participants were screened, reduced, and grouped according to Portuguese grammar, and then categorized according to their global meaning. The second stage included a frequency analysis of each attribute and the retention of the more frequent ones, insuring that a given attribute would only be retained in its positive or negative form (the one that was mentioned more frequently).

In order to select the attributes for analysis, we started by listing the attributes legibly written by participants (N = 738 attributes). Responses that could not be considered as attributes were excluded from this list (e.g., “to live out in the streets would be much worse”). Afterwards, attributes were checked for spelling mistakes and were grouped according to Portuguese linguistic criteria: singular and plural forms (e.g., amigo and amigos—friend and friends), gender (e.g., traumatizada and traumatizado–traumatized in female and male forms), etc. (for a similar strategy see [20,24]).

Subsequently, attributes were organized into categories according to their overall meaning (e.g., sad with unhappy; interested with attentive) by two Psychology researchers blind to the aims of the present study (for a similar procedure see [34,35]). Non-consensual cases were resolved by agreement between coders (80% consensus was achieved). Only the words reaching consensus between the coders were considered for further analyses.

The list resulting from the above procedures included 171 attributes. These attributes were then analyzed according to their frequency. All attributes referred to by participants less than twice were excluded. This was done once the frequency of these attributes was non-representative of the total attributes generated by participants. This procedure reduced the list to 84 attributes. Table 1 presents the list of these attributes and their respective frequency in each of the youth categories (residential care vs. mainstream) presented in the questionnaire.

**Table 1:** Attributes frequency by youth type.

	Frequency		
Attributes	Institutionalized youth	Mainstream youth	Total
Abandoned	4	3	7
Aggressive	10	2	12
Anxious	6	9	15
Bad-student	0	4	4
Calm	3	6	9
Cautious	2	0	2
Cherished	0	2	2
Clean	1	1	2
Committed	6	8	14
Competent	2	0	2
Confident	1	5	6
Conflicting	2	1	3
Confused	8	0	8
Courageous	2	0	2
Cultured	4	4	8
Dependent	1	1	2
Depressed	3	5	8
Deprived	2	4	6
Disobedient	1	1	2
Distrustful	6	0	6
Economically favored	0	2	2
Educated	7	14	21
Extroverted	3	4	7
Fighter	2	3	5
Friend	5	2	7
Frustrated	5	6	11
Funny	2	1	3
Good	1	1	2
Good-student	0	8	8
Happy	6	19	25
Hard-working	5	12	17
Healthy	2	6	8
Honest	0	3	3
Humble	7	8	15
Immature	1	2	3
Impatient	1	3	4
Impulsive	2	1	3
Independent	4	4	8
Insecure	14	7	21
Integrated	1	3	4
Intelligent	5	7	12
Introverted	15	3	18
Irresponsible	1	1	2
Irritable	3	0	3
Jealous	1	5	6
Lazy	2	4	6
Lonely	15	3	18
Loved	1	9	10
Low self-esteem	7	9	16
Misfit	7	3	10
Misunderstood	3	1	4
Motivated	1	5	6
Needy	18	7	25
Neglected	2	2	4
Nice	3	3	6
Obedient	0	2	2
Optimistic	1	4	5
Pessimistic	2	2	4
Poor	1	4	5
Presentable	1	4	5
Problematic	4	2	6
Protected	4	5	9
Rebel	4	2	6
Rebellious	38	13	51
Relaxed	0	8	8
Resistant	2	3	5
Responsible	2	7	9
Sad	29	16	45
Satisfied	1	2	3
Secure	2	2	4
Sensitive	13	1	14
Sociable	3	4	7
Sparing	1	3	4
Stable	0	6	6
Strong	4	2	6
Traumatized	9	5	14
Uncomfortable	2	1	3
Unmotivated	6	7	13
Unprotected	2	0	2
Unsatisfied	0	2	2
Unsociable	1	2	3
Unstable	5	2	7
Warm	2	2	4
With problems	4	0	4

These 84 attributes were then submitted to a final analysis. The antonyms of attributes were searched and eliminated (e.g., obedient vs. disobedient) in such a way that valence of attributes was kept balanced (the more frequent form was the one retained). The final list resulting from this procedure is presented in Table 2, and includes 68 attributes (34 positive and 34 negative).

**Table 2:** Final attributes list and assigned valence.

Attributes	Valence
Abandoned	Negative
Aggressive	Negative
Anxious	Negative
Calm	Positive
Cherished	Positive
Clean	Positive
Committed	Positive
Competent	Positive
Confident	Positive
Conflicting	Negative
Confused	Negative
Courageous	Positive
Cultured	Positive
Depressed	Negative
Deprived	Negative
Disobedient	Negative
Distrustful	Negative
Educated	Positive
Fighter	Positive
Friend	Positive
Frustrated	Negative
Funny	Positive
Good	Positive
Good-student	Positive
Happy	Positive
Hard-working	Positive
Healthy	Positive
Honest	Positive
Humble	Positive
Immature	Negative
Impatient	Negative
Impulsive	Negative
Independent	Positive
Insecure	Negative
Intelligent	Positive
Introverted	Negative
Irresponsible	Negative
Irritable	Negative
Jealous	Negative
Lazy	Negative
Lonely	Negative
Loved	Positive
Low self-esteem	Negative
Misfit	Negative
Misunderstood	Negative
Needy	Negative
Neglected	Negative
Nice	Positive
Pessimistic	Negative
Poor	Negative
Presentable	Positive
Problematic	Negative
Protected	Positive
Rebel	Negative
Rebellious	Negative
Relaxed	Positive
Resistant	Positive
Sad	Negative
Satisfied	Positive
Sensitive	Positive
Sociable	Positive
Sparing	Positive
Stable	Positive
Strong	Positive
Traumatized	Negative
Unmotivated	Negative
Warm	Positive
With problems	Negative

The attributes presented in Table 2 cover content areas like contextual circumstances (e.g., abandoned); individual competences (e.g., good student); emotions (e.g., happy) and behaviors (e.g., aggressive), as well as relational characteristics of the youth (e.g., friend).

## Discussion

This first study aimed at collecting attributes that laypeople as well as care professionals normally associate to youth in residential care. From an initial list of more than 700 attributes, 68 were selected to integrate the preliminary version of the SIEQ. This selection process was made taking into consideration the frequency of the attributes (i.e., referred at least twice by participants), as well as their valence (i.e., 34 positive and 34 negative attributes) and content (i.e., attributes cover areas such as contextual circumstances, individual competences, emotions and behaviors, and relational characteristics of the youth).

Overall, when asked to characterize youth in residential care respondents tended to use negative attributes. Specifically, the attributes most frequently referred by laypersons and professionals to describe this population were, among others, “rebellious”, “sad”, “needy”, “introverted”, “lonely” and “insecure”. Moreover, when compared to the attributes used to describe mainstream youth these same attributes where the ones that most differentiated these two groups of youth. Additionally, positive attributes such as “happy”, “relaxed”, “loved”, “good-student” or “hard-working”, were mainly used by respondents to describe mainstream youth. These findings confirm the negative social image people have about institutionalized youth.

Note that among these attributes, a large majority had already been identified in the literature as corresponding to the social image of youth in residential care (cf., [20,21,22,23,24,25]), but also to the attributes that youth in general identified in self-description tasks (i.e., “friend”, “intelligent”, “nice”, and “hard-working”) (cf., [31]).

The results of this first study were particularly useful to the subsequent phase of instrument development. Indeed, adjective checklists are classical and well-established procedures to generate items for psychological measurements, especially in the field of stereotypes (a proximal field of research to social images) [32,36]. The appropriateness of the attributes obtained for the development of an instrument to assess the social images of youth in residential care was further supported by their convergence with previous results regarding hetero and self-perceptions reported in the literature described above (e.g., [23]).

## Study 2

### Overview

Based on the results obtained in study 1, we developed a preliminary version of the SIEQ to examine the social images of youth in residential care. This questionnaire allows the systematic and quantitative evaluation of the image associated to this youth and the assessment of the positive or negative valence of this image. In this second study, we also analyzed the main psychometric properties of the SIEQ and present its final format to be used in future research. For this purpose, two samples were collected and two construct validity tests were performed, alongside with the assessment of other psychometric qualities of the SIEQ. The analyses of the attributes valence as well as an exploratory factorial analysis using principal axis factoring method were performed using the data from the first sample. In the second sample, a confirmatory factorial analysis was run over the factor structure obtained in the first sample and additional tests of factors’ sensitivity were also performed.

### Method

#### Participants: Sample 1

Participants (N = 296) voluntarily took part in this study. Participants age varied between 19 to 59 years old (*M* = 27.06, *SD* = 7.55) (5 participants did not reveal their age), with 90% being female, 82.8% single, 16.1% married or cohabiting, and 1.1% divorced (2 participants did not reveal their marital state). Around 15% of participants had 1 to 5 children with 0 to 26 years old (*M* = 7.73, *SD* = 6.38) (5 participants did not answer these questions). Regarding education, 62.5% of participants completed a major, 19.9% had a master degree or a PhD, 17.6% completed high school, and the remaining participants completed elementary school (2 participants did not reveal their education).

Regarding family income, 42.8% of participants earned between 1000 to 2000 Euros, 34% earned above 2000 Euros, while 21.9% earned below 1000 Euros (54 participants did not reveal their family income). Finally, 33.5% of participants worked in the area of youth in danger or risk or in youth protection services, social security, residential care institutions, schools with programs for youth in risk priority intervention, among others (3 participants did not answer this question).

#### Participants: Sample 2

Participants (N = 392) voluntarily took part in this study. Participants’ ages varied between 20 to 83 years old (*M* = 31.89, *SD* = 9.87) (9 participants did not reveal their age), with 86.2% being female. One quarter (25.3%) of the participants had 1 to 5 children (2 participants did not answer this question). Regarding education, 63.3% of the participants attended to or completed a major, and 21.4% attended or completed a master or had a PhD, and 13.8% of participants completed elementary or high school (6 participants did not reveal their education level).

Regarding family income, 45.4% of participants earned between 1000 and 2000 euros, 27.5% earned below 1000 euros, while, 20.8% earned between 2000 and 3000 euros, and 6.4% earned above 3000 euros (79 participants did not reveal their family income). Finally, 21.9% of participants worked in the area of youth in risk or in youth protection services, social security, institutions of residential care, schools with programs for youth in risk priority intervention, among others (2 participants did not answer this question).

#### Measures

The preliminary version of the SIEQ for youth in residential care was divided into three sections. In the first section data regarding the sociodemographic characteristics of respondents was collected, namely age, sex, income, education, and occupation. The second section was composed by 68 items corresponding to the 68 attributes obtained in study 1. Respondents were asked to evaluate the 68 attributes regarding their valence using a scale that ranged from 1 = very negative to 5 = very positive. In the third section, respondents were asked to rate the same 68 attributes regarding how much they described youth in residential care (1 = does not describe at all; 5 = describes a lot). The attributes were presented in a random order throughout the questionnaire.

#### Procedure and data analysis

The questionnaire was filled-out by a convenience sample, guaranteeing the confidentiality and anonymity of data collecting process. Participants were recruited via email (institutional and professionals’ mailing lists) and onsite during a scientific meeting. To recruit participants on-line we followed the same procedure as in study 1, and after obtaining the respective institutional authorizations, participants were contacted via email. The message briefly explained the aims of the study and asked for their collaboration. A link for the questionnaire was also provided. Following good practices in Internet data collection, participants that opened the link were further informed about the aims of the study and informed that they could abandon the questionnaire at any point simply by closing the web browser window (see [37]). After providing their informed consent to participate in the study (by clicking the “I Agree” option), participants were automatically directed to the SIEQ. At the end of the questionnaire, participants were presented with a debriefing text.

During registration at a scientific meeting about children at risk held at ISCTE-IUL in the end of 2014, attendants were asked if they would be willing to complete a questionnaire about their perceptions regarding youth. In the beginning of this “paper and pencil version”, participants were informed of the aims of the study and that they could return the questionnaire unfilled, since participation was fully optional. At the end of the questionnaire, participants were provided with a debriefing text. Participants were asked to return the SIEQ using the closed boxes that were available at the registration desk.

Data from sample 1 was fully collected using Qualtrics® platform (N = 272). Questionnaires of sample 2 were collected using Qualtrics® platform and during the scientific meeting (N = 120). Data collected via Qualtrics® platform were subjected to quality control using standardized procedures, namely checking for duplicated internet protocol (IP) addresses to prevent more than one questionnaire from the same IP [38].

In both versions of data collection, participants were guaranteed that the data analysis would be performed as a whole, and no individual analyses would be conducted. The study was executed in accordance with the existing Ethical Guidelines at Instituto Universitário de Lisboa (ISCTE-IUL), and approved by the Board of the Ethical Commission of the same institution.

Overall, there were no significant differences between the answers of participants to the SIEQ items collected via “Qualtrics” (M = 3.24; SD = .31) or “paper and pencil” version (M = 3.25; SD = .27) versions, *t* (390) = .47, *p* = .64, *d* = 0.03.

In order to obtain the final structure of the SIEQ, different analyses of the psychometric properties of the items composing this scale were performed. First, and using the first sample, the valence of the attributes to be included in the SIEQ was analyzed. After selecting the attributes with a clear positive or negative valence, a descriptive analysis of the items was performed. Thirdly, exploratory factorial analyses were conducted in order to obtain a stable structure as well as factors’ reliability. Finally, we analyzed the factors’ sensitivity to participants’ sociodemographic characteristics (i.e., gender, age, education, and professional status) as well as their intercorrelations.

Confirmatory factorial analyses were run using the second sample. A sensitivity analysis of the second-order factor was performed to test for differences regarding sociodemographic characteristics of the participants (gender, age, education, and professional status).

### Results

#### Analysis of the attributes’ valence

Using the data collected in sample 1, the valence of the attributes was analyzed using one-sample *t*-tests. All attributes with mean rating equal or inferior to 2 were taken as very negative or negative; those with mean rating equal or above 4 were taken as positive or very positive attributes. Attributes with ratings falling in the scale mid-point (i.e., 3) were excluded. As shown in Table 3, from the initial 68 attributes 14 were evaluated as negative or very negative, and 22 as positive or very positive. In this table, only the attributes that met the defined criteria are presented (a complete list of the valence test of all 68 attributes can be obtained from the first author).

**Table 3:** Descriptive statistics of SIEQ selected attributes and valence analyses.

Attributes	*M*	*SD*	Min	Max	Ske	Ske/s.e.	Kur	Kur/s.e.	Valence	One sample *t* test^a^		
										*df*	*t*	*p*
Abandoned	1,46	0,79	1	5	-0.65	-4.32	-0.71	-2.35	**-**	267	-11.20	.000
Neglected	1,60	0,91	1	5	-0.97	-6.47	0.30	1.00	**-**	265	-7.11	.000
Traumatized	1,65	0,90	1	5	-1.14	-7.59	1.18	3.93	**-**	267	-6.34	.000
Low self-esteem	1,75	0,97	1	5	-0.72	-4.79	0.21	0.69	**-**	267	-4.21	.000
Aggressive	1,79	0,93	1	5	-0.32	-2.16	0.14	0.45	**-**	266	-3.68	.000
Depressed	1,82	0,94	1	5	-0.50	-3.36	-0.09	-0.32	**-**	267	-3.12	.002
Problematic	1,83	0,90	1	5	-0.36	-2.42	-0.38	-1.25	**-**	267	-3.14	.002
Lonely	1,90	0,96	1	5	-0.59	-3.90	0.03	0.11	**-**	266	-1.79	.08
Misfit	1,93	0,90	1	5	-0.62	-4.13	-0.21	-0.68	**-**	267	-1.35	.18
Conflicting	1,94	0,95	1	5	-0.32	-2.13	-0.12	-0.39	**-**	266	-1.03	.30
Irresponsible	1,94	0,87	1	5	0.10	0.65	0.24	0.80	**-**	266	-1.20	.23
Unmotivated	1,97	0,97	1	5	-0.58	-3.85	0.05	0.18	**-**	267	-.51	.61
Sad	2,00	0,82	1	5	-0.62	-4.15	0.07	0.25	**-**	266	.000	1
Frustrated	2,02	0,87	1	5	-0.82	-5.49	0.34	1.14	**-**	267	.42	.67
Good-student	3,91	1,08	1	5	-0.11	-0.73	0.37	1.23	**+**	267	-1.31	.19
Protected	3,93	1,00	1	5	0.39	2.62	-0.20	-0.67	**+**	267	-1.22	.22
Satisfied	4,00	0,99	1	5	0.34	2.27	0.12	0.40	**+**	268	-.06	.95
Committed	4,02	0,87	1	5	-0.05	-0.33	0.61	2.03	**+**	267	.42	.67
Courageous	4,03	0,79	1	5	-0.08	-0.53	-0.01	-0.03	**+**	268	.69	.49
Sociable	4,03	0,79	1	5	0.03	0.17	0.06	0.19	**+**	266	.62	.54
Confident	4,04	1,02	1	5	0.57	3.78	-0.25	-0.84	**+**	267	.72	.47
Hard-working	4,07	1,01	1	5	-0.28	-1.84	0.76	2.54	**+**	267	1.21	.23
Clean	4,08	0,76	1	5	-0.25	-1.68	0.47	1.56	**+**	267	1.76	.08
Funny	4,08	0,74	1	5	0.12	0.78	0.28	0.95	**+**	268	1.80	.07
Intelligent	4,08	0,88	1	5	0.06	0.43	-0.17	-0.58	**+**	268	1.45	.15
Competent	4,11	0,79	1	5	-0.22	-1.46	1.22	4.07	**+**	264	2.26	.03
Fighter	4,13	0,89	1	5	-0.47	3.12	0.40	1.35	**+**	266	2.42	.02
Honest	4,17	0,92	1	5	-0.20	-1.34	1.06	3.53	**+**	268	2.99	.003
Cherished	4,19	0,85	1	5	0.42	2.83	-0.30	-1.00	**+**	266	3.62	.000
Nice	4,19	0,67	2	5	0.01	0.07	0.77	2.57	**+**	266	4.58	.000
Educated	4,20	0,91	1	5	-0.05	-0.36	0.77	2.58	**+**	268	3.70	.000
Good	4,22	0,72	2	5	0.24	1.60	0.40	1.34	**+**	268	5.51	.000
Happy	4,25	1,03	1	5	0.35	2.36	0.00	0.00	**+**	268	3.92	.000
Loved	4,37	0,84	1	5	0.48	3.23	0.13	0.42	**+**	267	7.31	.000
Friend	4,42	0,72	2	5	0.27	1.78	0.70	2.33	**+**	268	9.70	.000
Healthy	4,47	0,83	1	5	0.25	1.67	0.38	1.28	**+**	268	9.30	.000

Ske = Skewness; Ske/s.e. = ratio of skewness by standard error of skewness; Kur. = Kurtosis; Kur/s.e. = ratio of kurtosis by standard error of kurtosis.

^a^ Negative valence attributes were tested against the scale point “2”; positive valence attributes were tested against the scale point “4”. Items significantly equal or below/above each of these scale points were considered as truly negative or positive valence items.

#### Descriptive analysis of the attributes

After analyzing the valence of the attributes, we proceeded to a descriptive analysis of the 36 attributes with a clear valence. Note that we asked participants to think about youth in residential care and rate the attributes presented using a scale that ranged from 1 = does not describe at all to 5 = describes a lot. Table 3 presents the results of this descriptive analysis.

As it can be seen in Table 3, the mean ratings of the attributes range from *M*_*min*_ = 1.46 to *M*_*max*_ = 4.47. Standard deviation of attributes ranged from *SD*_*min*_ = 0.67 to *SD*_*max*_ = 1.08. Regarding item distribution, 12 negative attributes presented extreme negative skewed distributions (skewness by error of skewness ratio inferior to 2), and 7 positive attributes presented extreme positive skewed distributions (skewness by error of skewness ratio superior to 2) [39]. This means that participants tended to evaluate these attributes either in a very negative or positive way. Regarding kurtosis, we verified that 7 attributes (6 positive and 1 negative) presented leptokurtic distributions, and 1 negative attribute presented a platykurtic distribution. The remaining attributes presented a mesokurtic distribution. Overall, the remaining attributes followed the standards of the normal distribution.

#### Exploratory factorial analysis

After selecting the 36 attributes to include in the final version of the SIEQ, we analyzed the construct validity of this questionnaire. Using sample 1, we conducted a principal axis factorial analysis with *promax* rotation, since it was admissible that the resulting factors could have moderate intercorrelations. The number of factors retained in the extraction process was determined by analysis of the *scree plot*. Following good practices of factorial analysis [40], only items with loadings higher than .40 were retained in their respective factors. Table 4 presents a summary of the 30 items that were retained in the final analysis, as well as the loadings and *eigenvalues* for each factor, and their internal consistency (i.e., Cronbach *alphas*). The final solution revealed a high adequacy (KMO = .91), and three factors were retained, all with *eigenvalues* higher than 1. This factorial solution explained 52.16% of total variance. Item loadings in their respective factors were moderate to high, and factors presented a high internal consistency, with all items contributing moderately to highly to the internal consistency of the factors as is testified by the corrected item-total correlations.

**Table 4:** Exploratory factor analysis (principal axis factoring) of SIEQ items.

	F1	F2	F3	Corrected Item-total correlations
Items				
Sad and troublemaker youth (F1)				
Traumatized	**.82**	.03	.07	.73
Frustrated	**.80**	-.04	.26	.59
Sad	**.79**	.04	.04	.71
Depressed	**.76**	-.01	.23	.56
Low self-esteem	**.69**	-.06	.01	.66
Misfit	**.65**	.05	-.14	.71
Lonely	**.65**	.24	-.16	.63
Unmotivated	**.64**	-.08	-.06	.68
Neglected	**.63**	.18	-.22	.68
Problematic	**.56**	-.19	-.03	.61
Abandoned	**.53**	.13	-.06	.50
Conflicting	**.47**	-.14	-.19	.60
Aggressive	**.47**	-.24	-.11	.60
Self-competent youth (F2)				
Committed	-.07	**.77**	-.04	.68
Competent	.001	**.75**	.004	.69
Fighter	.16	**.74**	-.15	.54
Hard-working	-.10	**.69**	-.10	.60
Courageous	.09	**.63**	-.15	.47
Intelligent	-.13	**.62**	-.15	.55
Good	.11	**.59**	.19	.60
Honest	.002	**.48**	.22	.56
Friend	.12	**.47**	.28	.54
Educated	-.13	**.47**	.21	.57
Happy and nurtured youth (F3)				
Cherished	.05	-.15	**.84**	.66
Protected	.03	-.19	**.81**	.64
Loved	-.08	-.02	**.74**	.75
Satisfied	-.11	.07	**.67**	.73
Clean	.06	.23	**.53**	.52
Happy	-.27	.19	**.43**	.65
Healthy	.19	.29	**.43**	.41
Eigenvalue	9.87	3.83	1.95	—
Explained variance	32.92	12.75	6.49	—
Cronbach alpha	.90	.86	.86	—

The first factor, labeled “sad and troublemaker youth”, was composed by 13 items (*α* = .90; e.g., “traumatized”, “frustrated”, “sad”); the second factor, labeled “self-competent youth”, was composed by 10 items (*α* = .86; e.g., “competent”, “fighter”, “hard-working”); the third factor, labeled “happy and nurtured youth”, was composed by 7 items (*α* = .86; e.g., “cherished”, “protected”, “loved”). The correlations between these factors were moderate (f1-f2, *r* = .30, *p* < .001; f1-f3, *r* = .54, *p* < .001; f2-f3, *r* = .50, *p* < .001), which might indicate the presence of a second-order factor.

#### Descriptive analysis of factors

A descriptive analysis of the factors obtained was conducted. This analysis allowed us to verify the factors’ sensitivity regarding different sociodemographic characteristics of the participants, and further confirmed the psychometric qualities of the SIEQ (the “sad and troublemaker youth” factor items were reverse-scored to be in the same metric as the remaining factors). A MANOVA with SIEQ factors as dependent variables, and participants’ sex, education, having children, and working in the area of residential care as independent variables was conducted. The overall results of this analysis indicated that the scores in the SIEQ factors did not vary according to most of the sociodemographic characteristics of the participants (all *p*’s > .140). One exception was observed regarding participants that work in the area of youth at risk who perceived youth as more sad or troublemaker *M* = 2.26; *SD* = .67) than participants that do not work in this area, (*M* = 2.40; *SD* = .72), *F*(1,260) = 6.65, *p* < .01, *η*^*2*^_*p*_ = .03.

Regarding the factor scores *per se*, we compared the overall mean rating of each factor with the scale mid-point (3) using a one sample *t*-test. Results indicated that participants rated youth in residential care as more sad and troublemaker (*M* = 2.36, *SD* = .70), *t*(267) = -15.05, *p* < .001, *d* = 1.84, and as less happy and nurtured (*M* = 2.72, *SD* = .66) than the scale mid-point, *t*(267) = -7.01, *p* < .001, *d* = .86. However, this negative image was positively affected by the mean score in the self-competent factor (*M* = 3.27, *SD* = .52), *t*(267) = 8.42, *p* < .001, *d* = 1.03, which was significantly above the mid-point of the scale.

#### Confirmatory factorial analysis

To strengthen the assumption regarding the construct validity of the SIEQ, and based on the results obtained in the exploratory factorial analysis, three confirmatory models were tested using sample 2: (1) An uncorrelated factorial structure; (2) An intercorrelated factorial structure; (3) a second-order factorial structure (corresponding to our hypothesized model, given the results of the exploratory factorial analysis presented above).

**Fig 1:**
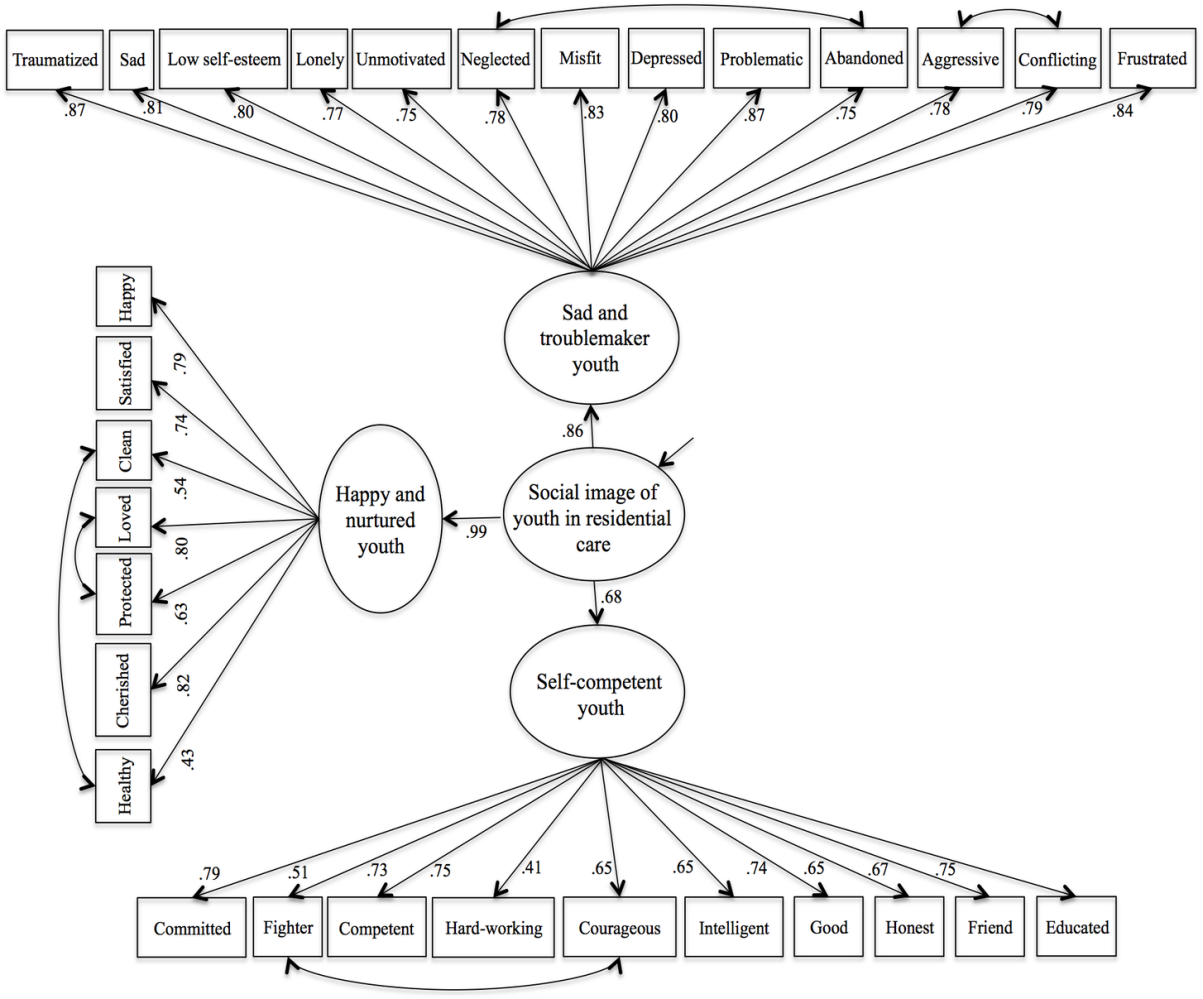
SIEQ second-order factorial structure.

The confirmatory models were run using Mplus 7 [41], using the Yuan-Bentler correction for nonnormality MLR estimator [42], and for the sake of model identification as well as to meet generally required specifications [43], on each first-order latent factors one indicator path loading was set to 1, and measurement errors paths to the indicator were all set to 1. Specifically, in the second-order model, the second-order component was set to 1. Both relative and absolute goodness of fit indexes of the models were obtained: the chi-square fit index (*χ*^2^); the relative chi-square fit index (*χ*^2^/df); the comparative fit index (CFI) [44]; the Tucker-Lewis fit index (TLI) [45]; the root mean square error of approximation (RMSEA) [46]; and the standardized root mean square residual (SRMR) [44]. Table 5 presents a summary of these analyses, and Fig 1 presents a graphical representation of the hypothesized second-order factorial structure.

**Table 5:** Summary of fit indexes for the SIEQ confirmatory models.

Models	*N*	*df*	*χ*^2^	*χ*^2^/*df*	*CFI*	*TLI*	*SRMR*	*RMSEA (CI)*
Model 1: Second-order factor	392	398	862.33	2.17	.91	.90	.15	.06 (.05;.06)
Model 2: First-order correlated factors	392	397	819.37	2.06	.92	.91	.08	.05 (.05;.06)
Model 3: First-order uncorrelated factors	392	400	1122,33	2.80	.86	.85	.22	.07 (.06;.07)

Based on the standards established in the literature for fit indexes (i.e., CFI and TLI indices greater than .90-.95; RMSEA lower than .08-.05; SRMR lower than .10-.08) [44,46,47,48,49], and as displayed in Table 5, models 1 and 2 presented acceptable fits, with moderate to high standardized regression paths between the items and their latent first-order factors, .39 < *λ* < .87, all *p* < .001. Model 1 presented moderate to high standardized regression paths between the first-order and the second-order factor, .68 < *γ* < .99, all *p* < .001 (see Fig 1). In Model 2, factor 1 significantly correlated with factor 2, *ϕ* = .43, *p* < .001, and with factor 3, *ϕ* = .76, *p* < .001. Factor 2 significantly correlated with factor 3, *ϕ* = .64, *p* < .001. As Model 3 presented the lowest fit indexes, we discarded this uncorrelated factorial structure as a good solution for the SIEQ.

Given the similarity between the fit indexes of Models 1 and 2, and the correlations between the factors found in the “first-order correlated factors” model, we reasonably assumed that the SIEQ is better represented by a second-order factorial structure. This means that the social image of youth in residential care is composed by three first-order factors (sad and troublemaker youth, self-competent youth, and happy and nurtured youth) with a second-order factor that corresponds to the general image of these youth (see Fig 1). The latent factors reliability was high as computed by the H reliability coefficient [50]: sad and troublemaker youth, *H* = .96; self-competent youth, *H* = .91; happy and nurtured youth, *H* = .89; second-order factor, *H* = .98. This second-order model also followed the general structure obtained in the exploratory factor analysis, and supported our prediction that the factorial intercorrelations could be signaling a second-order dimension.

#### Descriptive analysis of second-order factor

Since the descriptive analysis of the first-order factors was presented previously, and based on the results of the confirmatory factorial analysis, we ran descriptive and sensitivity analyses for the second-order factor of “general social image of youth in residential care”. These analyses showed that this factor was globally evaluated in a negative way, *t* (391) = -6.22, *p* < .001, *d* = .63, below the scale’s mid-point (*M* = 2.84, *SD* = .52). Also, the sensitivity analyses showed that this factor varied according to the working area of the participants (work in the area of youth at risk vs. not), *t* (388) = -3.42, *p* < .001, *d* = .35. More specifically, respondents not working in the area reported a less negative social image of youth (*M* = 2.88, *SD* = .54) than participants that work in the area (*M* = 2.67, *SD* = .40). Also, participants with children had a more negative image of youth in residential care (*M* = 2.69, *SD* = .42) than participants without children (*M* = 2.89, *SD* = .54), *t* (388) = -3.28, *p* < .001, *d* = .33. The second-order factor was not sensitive to the remaining sociodemographic characteristics of the sample, namely gender and education (all *p*’s > .09).

### Discussion

In this second study, we developed and determined the construct validity and reliability of the SIEQ. Indeed, after selecting the attributes that would figure in a preliminary version of the present scale, in this second study we developed the final measurement instrument including the attributes that clearly present a positive or a negative valence. This final instrument composed by 17 positive and 13 negative attributes was tested in two independent samples of participants. In the first sample, a principal axis factoring analysis revealed a three-factor structure, with moderate to high inter-factorial correlations that retained 30 of the initial 36 items.

The social image conveyed by these factors presented youth in residential care as “sad and troublemaker”, as less “happy and nurtured”, but as “self-competent”. This three-folded image follows other studies in this area that emphasizes the atypical nature and the ambiguity of the impact of residential care on the social image of institutionalized youth [20,51].

In a second sample, this three-factor structure was tested with a confirmatory factorial analysis. Three models were analyzed: a non-correlated three-factor structure, a correlated three-factor structure, and a second-order factor structure with three first-order factors. This last structure was our hypothesized model since it brought us closer to our exploratory factorial analysis. The results of the confirmatory factorial analysis supported the correlated and the second-order factorial structures, and as such a second-order factorial structure was retained. Therefore, and in addition to depicting the social image of youth in residential care as a mixture of positive and negative attributes distributed by three different factors, the SIEQ allows obtaining a general image that is structured by these factors. Indeed, the overall social image of youth in residential care was evaluated in a negative way, below the mid-point of the scale.

Moreover, additional analyses regarding the sensitivity of SIEQ to different sociodemographic characteristics of our sample were performed. These analyses showed that the second-order factor is sensitive to respondents’ working area, that is, participants that work in the area of youth at risk tended to have a more negative overall image of youth in residential care than participants who did not deal with this population at a regular basis. Also, participants with children had a more negative image of youth in residential care than participants that did not have children.

## Conclusions

Literature has been suggesting to the fact that residential care is associated to an overall negative social image with severe consequences for youth well-being and identity [22,52,53]. However, and to the best of our knowledge, there are no measurement instruments tailored to analyze in a quantitative and systematic way the social image of youth in residential care. In order to address this shortcoming, and following the procedures of other studies in this area [30,31], we conducted two studies that present the development and validation of the SIEQ.

In the first study, we identified a set of 84 attributes that characterize youth in residential care, from which 68 were selected to compose the SIEQ. In the second study, these latter attributes were tested using two different samples. In the first sample, the valence of these items was tested and from the 68 initial attributes 36 with a clear positive or negative valence were retained for the final version of the SIEQ. Also with this same sample, we tested the factorial structure and internal consistency of the SIEQ in order to assure its construct validity and reliability.

The final structure of the SIEQ presents three intercorrelated factors that describe the social image regarding youth in residential care. These factors have high internal consistency, with one factor depicting youth as sad and troublemaker; a second factor depicting youth as self-competent; and finally a third-factor presenting youth as happy and nurtured. The first and the third factor globally presented negative mean ratings (below the mid point of the scale). The second factor was globally positive (is evaluated above the mid point of the scale). These results presented a mixed social image of youth in residential care, that is, an image composed by positive and negative attributes, and consistent with results that have been reported in the literature (e.g., [20,51]).

This factorial structure was further assessed in a second sample via confirmatory factorial analysis. Three models were tested and two of them revealed adequate fits–the correlated factors and the second-order factor models. We opted for the second-order structure since it better corresponded to our framework regarding the social image of youth in residential care, and was in line with the results obtained in the exploratory factorial analysis. Note that this second-order factor presented a negative scoring in our sample, below the mid-point of the response scale.

In a nutshell, we have presented preliminary evidence of the good psychometric qualities of the SIEQ and of its potential as an adequate measurement instrument to analyze the social image of youth in residential care. Thus, the implications of the SIEQ for research and practice are numerous. To begin with, this instrument allows research to take one step further by measuring in a systematic and quantitative way the social images of youth in residential care. This enables a reliable procedure to tap the images used by both lay-people and care professionals to describe youth in residential care, and to use this knowledge to train specialized personnel working in this area. Additionally, the scores from the SIEQ might help the comparison process of social images with youth self-images and meta-images, supporting successful interventions with this population. Finally, these same scores allow comparisons with other groups of youth, namely those living in mainstream families.

### Limitations and future studies

The studies presented in this article do not go without limitations. First, respondents living in urban areas, highly educated, and mostly female compose the samples used in the different studies. Future studies should test the SIEQ in more heterogeneous samples. Second, further investigation is required regarding the differences between the social images of youth in residential care held by laypeople and professionals. Indeed, while some studies indicate that the images of children and youth in care are the same for residential care workers and laypeople (e.g., [20]), others present conflicting evidence (e.g., [21]). In future studies, the SIEQ could be used to systematically and quantitatively investigate differences in the social images held by professionals and laypeople. This is particularly important as there is literature indicating the particularly negative impact for youth in care to feel stigmatized by people directly working with them [54]. Third, and like any other self-report instrument, SIEQ responses may have been vulnerable to social desirability. In order to surpass these limitations, future studies should focus on testing the psychometric qualities of SIEQ, adding for example to the completion protocol a lie scale to control for social desirability. Forth, other psychometric qualities should be tested, like convergent validity with other existing measures of social image of youth in residential care, and predictive validity. Regarding this latter type of validity, future studies could analyze the capability of the SIEQ to predict adjustment of youth in residential care in mainstream society. Additionally, the SIEQ should be tested using a larger sample to increase the ratio of number of participants-per item thus guaranteeing a stronger construct validation of this measure. Finally, the SIEQ should be tested cross-culturally in order to analyze the applicability of its present attributes and its factorial structure to other cultural contexts and to respondents with different cultural backgrounds.
